# Supplementary material for: Structure in the variability of the basic reproductive number (R0) for Zika epidemics in the Pacific islands
Source: eLife. 2016 Nov 29;5:e19874. doi: 10.7554/eLife.19874 (PMC5262383; doi:10.7554/eLife.19874)
Supplement: Supplementary file 1. — DOI: http://dx.doi.org/10.7554/eLife.19874.031 [file elife-19874-supp1.zip › Codes_for_SSM/READ ME.pdf]

Structure in the variability of R_0 for Zika epidemics in the Pacific islands

C Champagne, DG Salthouse, R Paul, VM Cao-Lormeau, B Roche, B Cazelles

Using SSM software to reproduce the calculations

1. Install SSM software on your machine as indicated in https://github.com/JDureau/ssm
The complementary module "SSM predict" also needs to be installed (https://github.com/sballesteros/ssm-predict)

2. In order to run one model, four elements are needed:

a. Data files in csv format
b. Priors distributions in json format
c. An initialisation file in json format
d. The json file containing the model, called "ssm.json"

These 4 elements are provided for each model, in a file with the corresponding names. The initialisation file provided here is the result of a previous simplex run (called theta_simplex.json) but one can start with other initial values (also put in a file named theta_simplex.json for using with the given code).

3. Execute the R code "pmcmc_kmcmc_simulations.R".
This files enables to compile the model, run KMCMC and PMCMC algorithm and do a simulation of the model (using our estimated values).

The PMCMC part is to be used when convergence is reached with KMCMC algorithm, which might sometimes require more than 5 KMCMC steps.
The PMCMC part is computationally intensive and may require a cluster (when the number of particles is 10,000).

We provided our estimates for Pandey model in Tahiti as an example, in the "pmcmc-result" file: they comprise the last PMCMC chain (trace_0.csv), the filter trajectory (X_0.csv), the last set of parameters of the chain (mle.json) and the simulation of the model (X_simul.csv)

4. All the calculation is done, and the outputs can be plotted using for example the R code "plotting".
