# Supplementary figures and images for: Structure in the variability of the basic reproductive number (R0) for Zika epidemics in the Pacific islands
Source: eLife. 2016 Nov 29;5:e19874. doi: 10.7554/eLife.19874 (PMC5262383; doi:10.7554/eLife.19874)

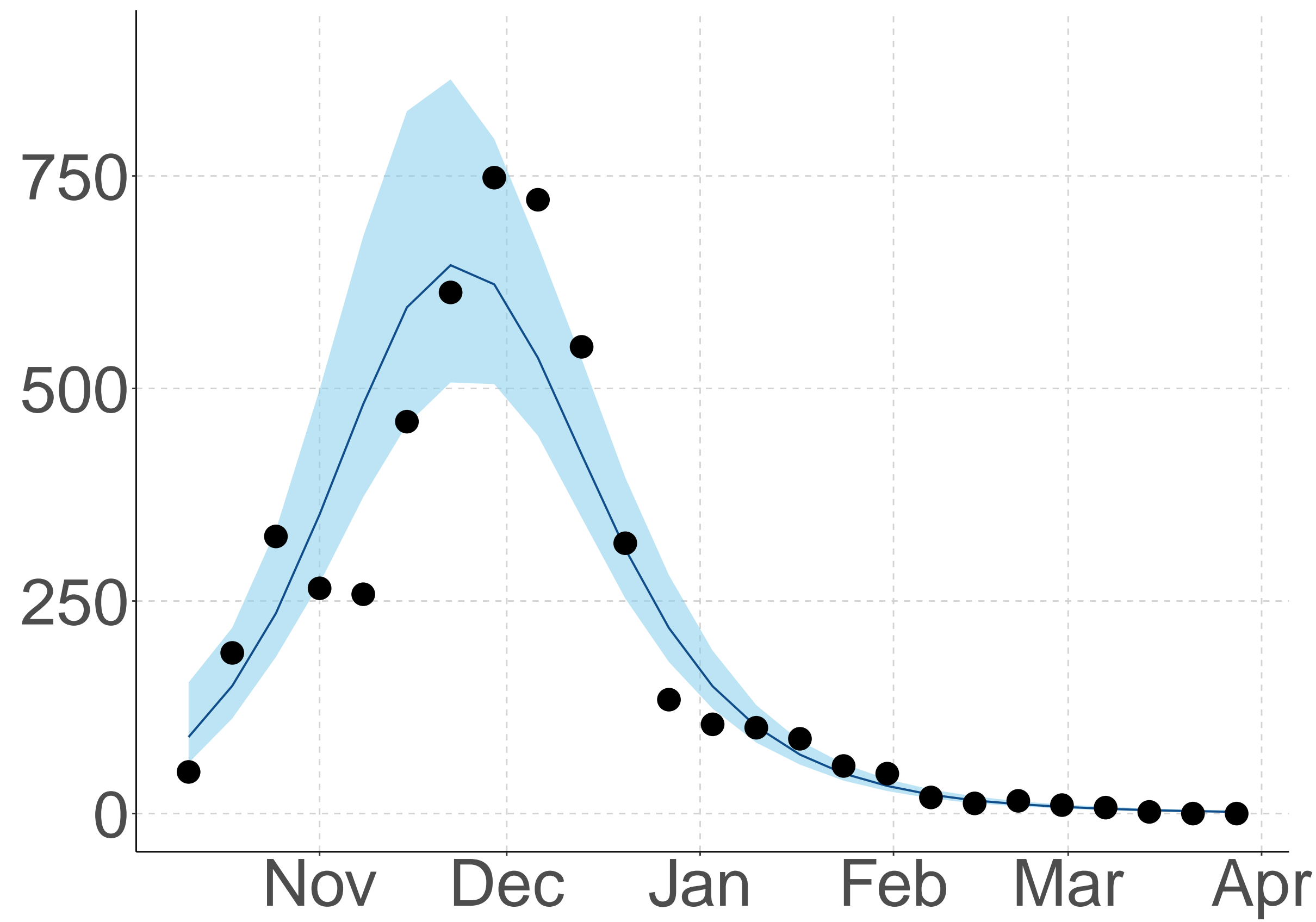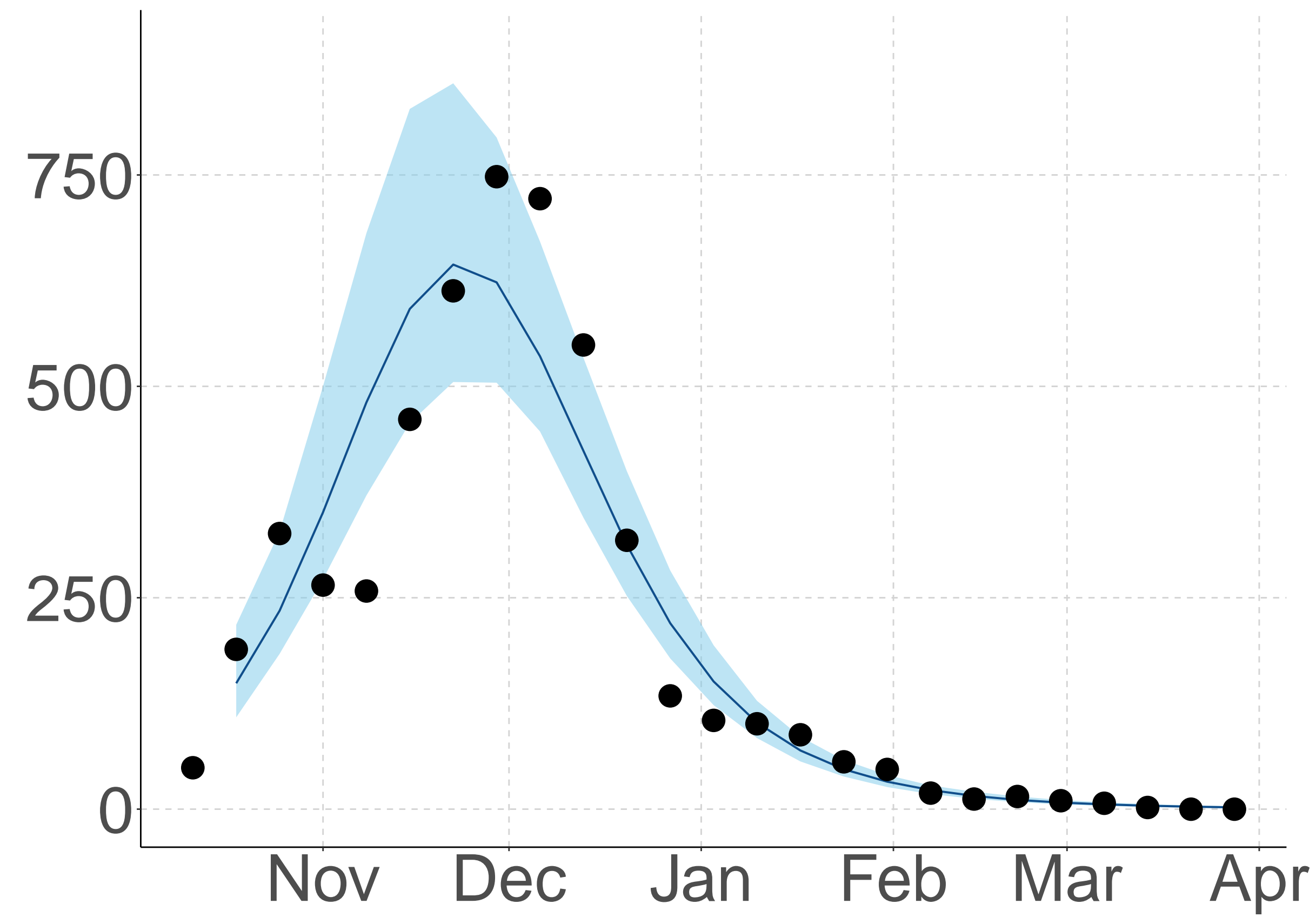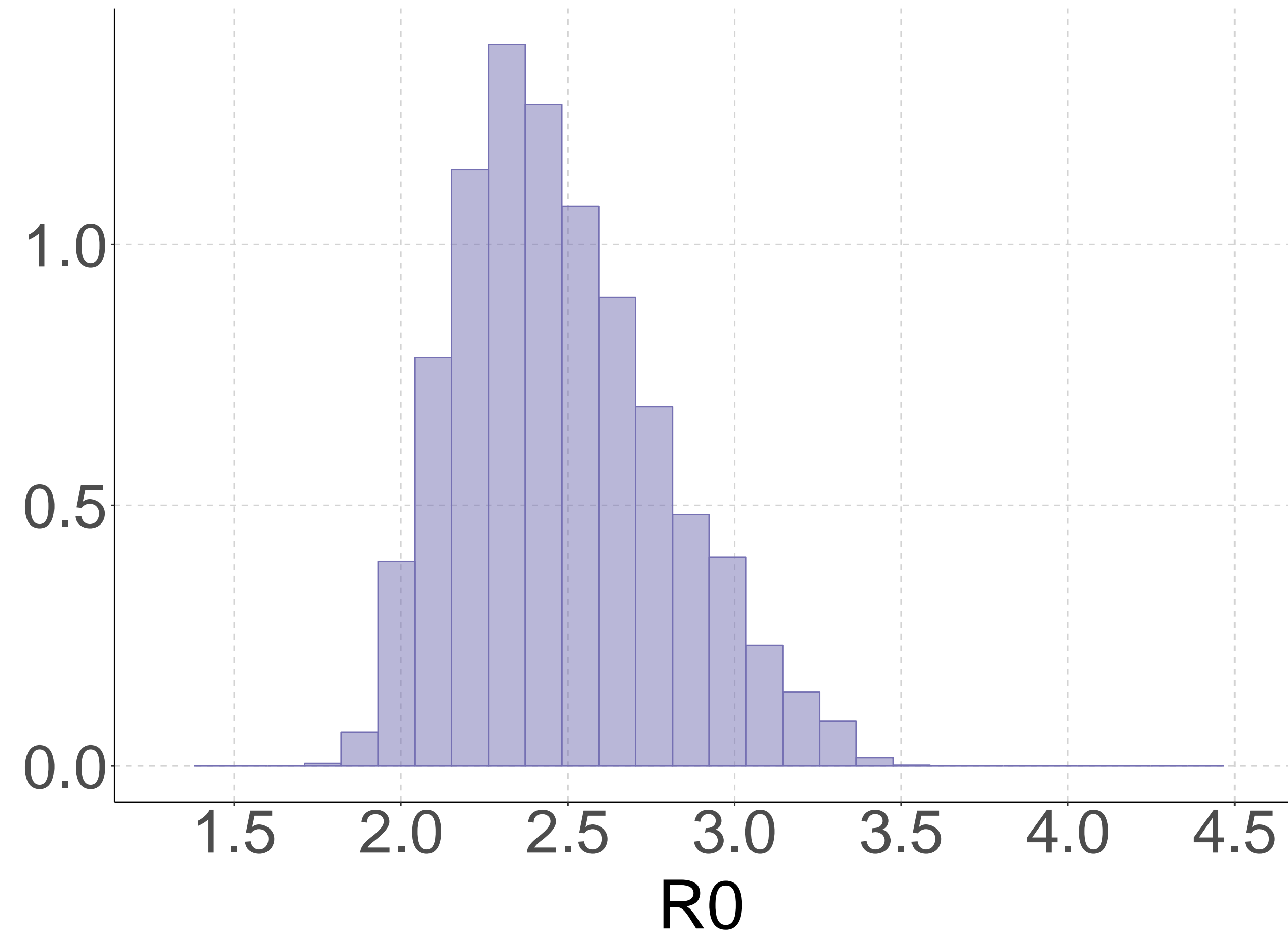

Supplement: Supplementary file 1. — DOI: http://dx.doi.org/10.7554/eLife.19874.031 [file elife-19874-supp1.zip › Codes_for_SSM/Graphes/main_tahiti_pandey.pdf]
